# DNA-launched RNA replicon vaccines induce potent anti-SARS-CoV-2 immune responses in mice

**DOI:** 10.1038/s41598-021-82498-5

**Published:** 2021-02-04

**Authors:** Inga Szurgot, Leo Hanke, Daniel J. Sheward, Laura Perez Vidakovics, Ben Murrell, Gerald M. McInerney, Peter Liljeström

**Affiliations:** Department of Microbiology, Tumor and Cell Biology, Karolinska Institutet, Stockholm, Sweden

**Keywords:** SARS-CoV-2, DNA vaccines

## Abstract

The outbreak of the SARS-CoV-2 virus and its rapid spread into a global pandemic made the urgent development of scalable vaccines to prevent coronavirus disease (COVID-19) a global health and economic imperative. Here, we characterized and compared the immunogenicity of two alphavirus-based DNA-launched self-replicating (DREP) vaccine candidates encoding either SARS-CoV-2 spike glycoprotein (DREP-S) or a spike ectodomain trimer stabilized in prefusion conformation (DREP-S^ecto^). We observed that the two DREP constructs were immunogenic in mice inducing both binding and neutralizing antibodies as well as T cell responses. Interestingly, the DREP coding for the unmodified spike turned out to be more potent vaccine candidate, eliciting high titers of SARS-CoV-2 specific IgG antibodies that were able to efficiently neutralize pseudotyped virus after a single immunization. In addition, both DREP constructs were able to efficiently prime responses that could be boosted with a heterologous spike protein immunization. These data provide important novel insights into SARS-CoV-2 vaccine design using a rapid response DNA vaccine platform. Moreover, they encourage the use of mixed vaccine modalities as a strategy to combat SARS-CoV-2.

## Introduction

The severe acute respiratory syndrome coronavirus 2 (SARS-CoV-2) emerged as the causative agent of COVID-19 in late 2019^1,2^. The disease pathology ranges from asymptomatic infection to severe acute respiratory distress and death^3,4^. Because the human population is immunologically naïve to SARS-CoV-2, the virus spread rapidly inducing a global pandemic. As of December 16, 2020, more than 73 million people globally have been infected with more than 1.6 million deaths (according to European Centre for Disease Prevention and Control).

The development of an effective and versatile vaccine platform is central to combat COVID-19 and to meet potential future threats. An ideal vaccine platform would be potent, rapid to produce, stable and affordable. The platform should also be easily, and rapidly adaptable in case new SARS viruses or new variants of SARS-CoV-2 will emerge so that novel threats can be rapidly met.

DNA-launched self-amplifying replicon RNA vectors (DREP) are very well suited for rapid vaccine development because they can encode any antigen of interest, require a minimal dose compared to conventional DNA vaccines and provide platform manufacturing technologies in which upstream supply chains and downstream processes are the same for each product^5,6^.

**Figure 1:**
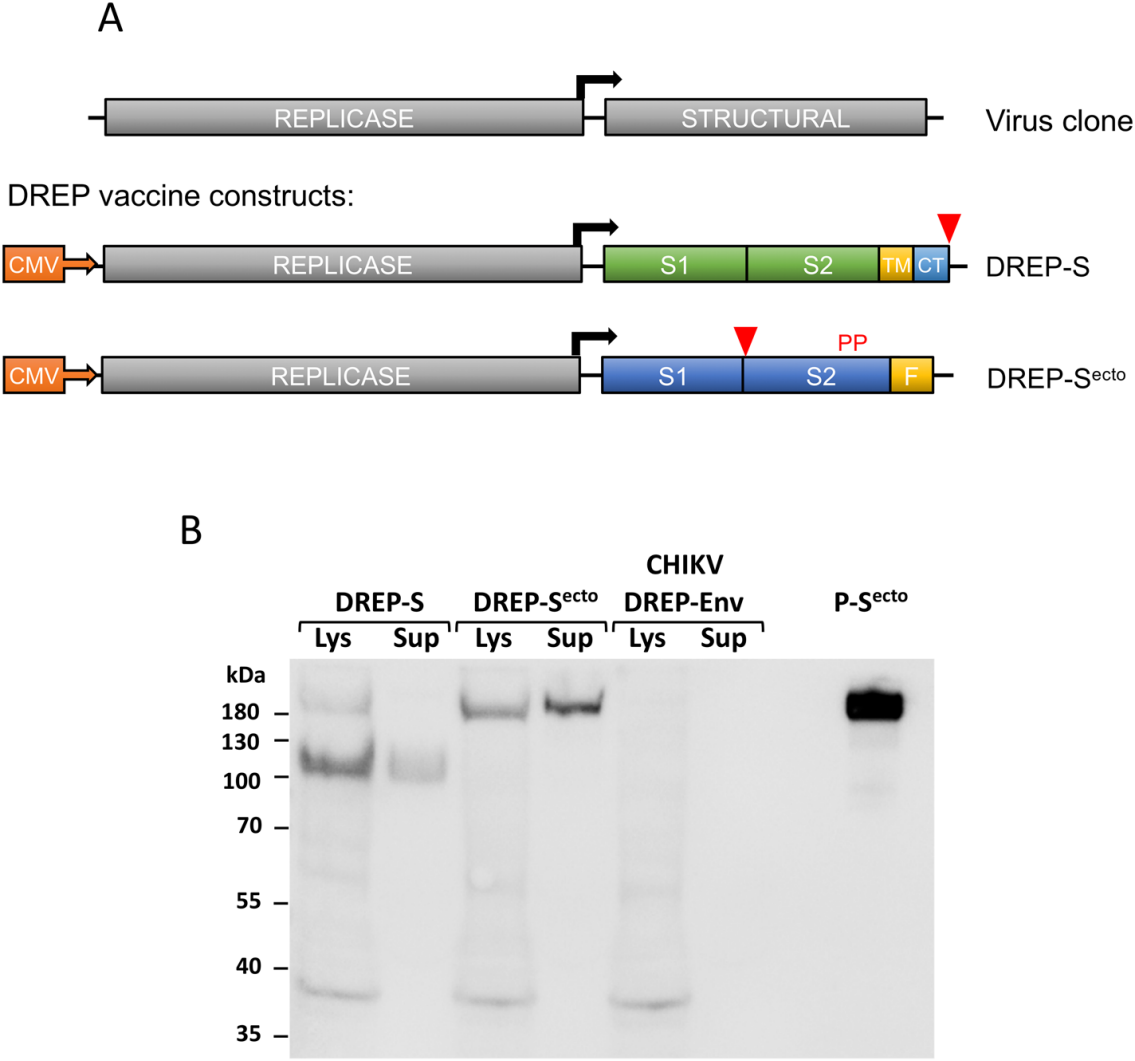
Schematic illustration of the DREP vaccine candidates encoding SARS-CoV-2 spike (S) protein and analysis of expression form the vaccine constructs. (**A**) Top, an infectious clone of an alphavirus showing the replicase and structural coding regions placed under the viral subgenomic promoter (black arrow). To target SARS-CoV-2 we replaced the alphavirus structural genes with the genes encoding the spike protein of SARS-CoV-2. The DREP-S construct codes for the SARS-CoV-2 spike protein with an 18-aa deletion in the cytoplasmic tail region, while S^ecto^ codes for the prefusion-stabilized spike ectodomain with a T4 fibritin (foldon, F) trimerization domain, two stabilizing prolines (PP) and a mutated furin cleavage site; TM, transmembrane domain; CT, cytoplasmic tail. Deletions are indicated by red arrows. (**B**) HEK293T cells were transfected with either DREP-S or DREP-S^ecto^ construct, or DREP encoding CHIKV spike glycoproteins as negative control. Cell lysates (Lys) and supernatants (Sup) were analyzed by Western blot using alpaca anti-spike serum. S^ecto^ protein was used as positive control.

Our DREP platform is based on the alphavirus genome derived from Semliki Forest virus (SFV), and encodes the genes for the viral RNA replicase but lacks the genes coding for the structural proteins of the virus^5,7^. Instead the DREP carries the gene(s) encoding the antigen of interest (Fig. 1)^7^. When delivered into cells during vaccination, the DNA-launched RNA molecule replicates in the same fashion as it would during an alphavirus infection with the important distinction that new viral particles are not formed^5^. Replicon vectors have inherent adjuvant properties due to their self-amplifying activity which generates single-stranded and double-stranded RNA intermediates that stimulate different pattern recognition receptors (PRR) including the endosomal TLR3, TLR7, and TLR8^8,9^ or the cytoplasmic MDA-5^10^, RIG-I^10,11^ and PKR^12,13^. This results in induction of a type I interferon (IFN) response, translational shutoff of a host cell and induction of apoptosis thereby promoting antigen presentation on MHC class I molecules and cross-priming^11,14–16^. Induction of apoptosis of the transfected cells eliminates the unlikely event of chromosomal integration and the risk of subsequent malignant transformation^17^. Thus, DREP platform combines the safety profile of conventional plasmid DNA vaccines that proved safe in multiple clinical trials, with adjuvant features of viral vectors. Additionally, in contrast to mRNA vaccine candidates, the DREP platform is highly stable and does not require a cold-chain^5^.

To elicit and evaluate immune responses against the SARS-CoV-2 spike, we inserted the genes encoding two different variants of the spike glycoprotein into DREP. The spike protein of SARS-CoV-2 mediates receptor binding and entry into target cells, which makes it an attractive vaccine antigen. For a vaccine intended to generate high-quality neutralizing antibody responses, delivering a conformationally correct protein that displays native surface epitopes is critical^18^. Previous studies on SARS or MERS have elucidated the need to stabilize coronavirus spike proteins in their pre-fusion conformation in order to serve as a vaccine immunogen^19,20^.

Here, we evaluate and compare, in a preclinical C57BL/6J murine model, the immunogenicity of two DREP vaccine candidates encoding either SARS-CoV-2 (Wuhan-Hu-1) spike glycoprotein only containing an 18-aa deletion (Δ18) in the cytoplasmic tail or a pre-fusion stabilized spike ectodomain with a C-terminal T4 trimerization domain, and two stabilizing proline substitutions in the C-terminal S2 fusion machinery^19–21^. We characterized both the humoral and cellular responses as well as the neutralization capacity of a pseudotyped SARS-CoV-2 virus in different prime-boost immunization regimens.

## Materials and methods

### DREP SARS-CoV-2 vaccine candidates

DREP SARS-CoV-2 vaccine constructs were made by cloning the sequences encoding S or S^ecto^ variants of SARS-CoV-2 spike protein into the Semliki Forest virus (SFV) DREP plasmid vector backbone^7^ using BamHI and SpeI restriction sites. The S construct encodes the surface glycoprotein of SARS-CoV-2 (Wuhan-Hu-1) with an 18-aa deletion in the cytoplasmic tail (Δ18). S^ecto^ construct codes for the SARS-CoV-2 prefusion-stabilized spike ectodomain. This version ends at glutamine Q1208 of the QHD43416.1 (GenBank accession number) gene sequence followed by a GSG linker and a T4 fibritin (foldon) trimerization motif. The pre-fusion state of the spike glycoprotein was stabilized by proline substitutions of K968 and V969. Additionally, the furin cleavage site between S1 and S2 subunits was modified to produce an uncleaved S protein. The synthesis of the constructs with the appropriate restriction sites was ordered from Twist Bioscience. Both spike variants were codon optimized for human expression and the constructs were confirmed by sequencing. Plasmid DNA of the different DREP SARS-CoV-2 vaccine candidates was purified from bacterial cultures using the EndoFree Plasmid Maxi or Giga Kit (QIAGEN) and the concentration and purity was measured on a NanoDrop One (Thermo Fisher Scientific).

### Recombinant proteins

The plasmid for expression of the SARS-CoV-2 prefusion stabilized spike ectodomain 1 was kindly provided by Jason McLellan^22^. This plasmid was used to transiently transfect expiCHO cells using the ExpiFectamine CHO Transfection Kit (Gibco). The spike ectodomain was purified from filtered supernatant on HisPur Ni–NTA resin (Thermo Fisher Scientific), followed by size-exclusion chromatography on a Superdex 200 in 5 mM Tris pH 8, 200 mM NaCl.

The RBD domain (RVQ-VNF) of SARS-CoV-2 was cloned upstream of a Sortase A recognition site (LPETG) and a 6xHIS tag. This plasmid was used to transiently transfect FreeStyle 293F cells using the FreeStyle MAX reagent. The protein was purified from filtered supernatant on HisPur Ni–NTA resin followed by size-exclusion chromatography on a Superdex 200 in 5 mM Tris.

### Western Blot

HEK293T cells (ATCC-CRL-3216) were transfected with either DREP-S or DREP-S^ecto^ construct, using Lipofectamine 3000 (Invitrogen) and cells and supernatants were harvested 24 h later. As a negative control, we used cells transfected with DREP encoding chikungunya virus (CHIKV) envelope protein (Env)^23^. Cells were then lysed with a buffer containing 20 mM HEPES pH 7.4, 0.5 M sodium chloride, 110 mM potassium acetate, 2 mM magnesium chloride, 1.0% Triton X-100, 0.5% sodium deoxycholate, 0.1% Tween 20 and protease inhibitors cocktail (Thermo Fisher Scientific), and cell debris was removed by centrifugation (4 °C/10 min/14,000 rpm). Both cell lysates and cell culture supernatants were then mixed with sample buffer (Thermo Fisher Scientific), heated for 10 min at 90 °C and run on a 4–12% SDS-PAGE gel. Proteins were transferred to a nitrocellulose membrane using a semi-dry blotting system (Bio-Rad) and membrane blocking was performed for 1 h at room temperature (RT) using 5% (W/V) non-fat milk powder (Sigma) in Tris-buffered saline (TBS, Thermo Fisher Scientific). The expression from DREP constructs was analyzed by Western blot using anti-spike serum from an immunized alpaca (1:2,000) followed by incubation with anti-llama IgG secondary antibody conjugated with horseradish peroxidase (HRP) at 1:10,000 (Abcam #ab112786). The membrane was developed using an ECL Plus Western blotting detection system (Thermo Fisher Scientific). S^ecto^ protein (50 ng) was used as positive control.

### Animals and immunizations

The vaccine candidates were tested in 6–8 weeks old female C57BL/6J mice (Charles River, Germany). Animals were kept at the Astrid Fagraeus laboratory, Karolinska Institutet, in accordance with the recommendations of the Swedish Board of Agriculture. The protocol was approved by the local ethics committee, Stockholms norra djurförsöksetiska nämnd, permit number 18033-18, and all animal procedures were carried out according to the approved guidelines, and in compliance with the ARRIVE guidelines (Animal Research: Reporting of In Vivo Experiments). Prior to immunization all mice were anesthetized by isoflurane inhalation. DREP vaccines were administered by intradermal (i.d.) needle injection followed by electroporation (EP), as described previously^24^. A total volume of 2 times ~ 20 μl was injected i.d. containing 10 μg DREP DNA with a 30-gauge insulin syringe (BD Micro-Fine). Then, a needle array electrode with two parallel rows of four 2 mm pins was placed at the injection spot, and 2 pulses of 1.125 V/cm for 50 μs followed by 8 pulses of 275 V/cm for 10 ms were applied. The recombinant SARS-CoV-2 spike protein (S^ecto^, 10 μg) was diluted in sterile PBS, emulsified in AddaVax (InvivoGen) adjuvant at a 1:1 protein to adjuvant volume ratio 30 min before immunization, and a volume of 50 μl was injected intramuscularly (i.m.) in the left hind leg gastrocnemius muscle.

### SARS-CoV-2 ELISA

Maxisorp 96-well plates (Nunc, Odense, Denmark) were coated with 2 μg/ml of either prefusion-stabilized spike protein (S^ecto^) or RBD antigens. After 24 h at 4 °C, plates were washed with PBS supplemented with 0.05% Tween (Sigma-Aldrich; PBS-Tween) and blocked with 5% skim milk in PBS for 2 h at room temperature. Sera were diluted in PBS-Tween (0.05%) at a 1:100 starting dilution and then serially diluted until a 1:218,700 dilution. 50 µl of serum was added per well and plates were then incubated at 4 °C overnight. The following day, the plates were washed three times with PBS-Tween (0.05%) before a HRP-conjugated goat anti mouse-IgG (Southern Biotechnology) or IgG1, or IgG2c (both Invitrogen) was added at a 1:5000 dilution in PBS-Tween, 50 µl/well. After 2-h incubation at RT, plates were washed six times with PBS-Tween (0.05%) and developed using 50 µl of o-phenylenediamine dihydrochloride substrate (Sigma Fast, Sigma Aldrich). After 15 min at RT, the reaction was stopped with 25 µl 1 M HCl and the optical density (OD) was read at 490 nm using a Victor2 Microplate Reader (Wallac). For calculation of endpoints titers, a cutoff value based on the OD490 values from the naive controls was determined (2 times average from naïve control sera at the lowest dilution). Titers were determined by interpolating the point where the sigmoid curve reaches the cutoff value using the GraphPad Prism 7 software^25^. EC_50_ titers were calculated by fitting a logistic curve and interpolating the value midway between the plate minimum and maximum using Prism 7 (GraphPad Software).

### Neutralization assay

Pseudotyped neutralization assays were adapted from protocols previously validated to characterize the neutralization of HIV^26^ but with the use of HEK293T-ACE2 cells, as previously described^21^. All cell lines were cultured in a humidified 37 °C incubator (5% CO_2_) in Dulbecco’s Modified Eagle Medium (Gibco) supplemented with 10% Fetal Bovine Serum and 1% Penicillin/Streptomycin, and were passaged when almost confluent using 1x Trypsin–EDTA. All cell lines tested negative for mycoplasma by PCR. All serum samples were heat inactivated at 56 °C for 60 min to inactivated complement. Pseudotyped lentiviruses displaying the SARS-CoV-2 spike protein (harboring an 18 amino acid truncation of the cytoplasmic tail) and packaging a luciferase reporter gene were generated by the co-transfection of HEK293T cells using Lipofectamine 3000 (Invitrogen) per the manufacturer’s protocols. Media was changed 12–16 h after transfection, and pseudotyped viruses were harvested at 48- and 72 h post transfection, filtered through a 0.45 μm filter, and stored at -80 °C until use. Pseudotyped viruses standardized to a multiplicity of infection that generates ~ 100,000 relative light units (RLUs) were incubated with serial dilutions of serum for 60 min at 37 °C in a 96-well plate, and then 15,000 HEK293TACE2 cells were added to each well. Plates were incubated at 37 °C for 48 h, and luminescence was then measured using Bright-Glo (Promega) per the manufacturer’s protocol, on a GM-2000 luminometer (Promega). ID_50_ titers were calculated by fitting a logistic curve and interpolating the reciprocal serum dilution at which RLUs were reduced by 50% relative to control wells in the absence of serum using Prism 5 (GraphPad Software).

### Isolation of splenocytes

Isolation of splenocytes was performed as described previously^25^. Briefly, freshly isolated mouse spleens were mashed though 70-μm cell strainers. Cells were subsequently washed by adding 10 ml of complete RPMI medium [RPMI 1640 supplemented with 10% fetal bovine serum (FBS), 2 mM l-glutamine, 100 U/ml penicillin and 100 μg/ml streptomycin] (Thermo Fisher Scientific) and centrifuged (350×*g* at RT). Cells were then resuspended in 1 ml of red blood cell lysis buffer (Sigma-Aldrich) for 2 min. Lysis was stopped by addition of 10 ml of complete RPMI medium, and the cells were washed as above before they were resuspended in complete RPMI medium. Cells were quantified with the Countess II cell counter (Thermo Fisher Scientific).

### IFN-ELISpots

Assessment of the IFN-γ T cell response was performed using the Mouse IFN- ELISpot^PLUS^ kit (Mabtech) following the manufacturer’s instructions. Briefly, anti-IFN-γ pre-coated plates were blocked with RPMI + 10% FBS for at least 30 min. 2 × 10^5^ freshly isolated splenocytes were added per well in triplicates with SARS-CoV-2 peptide pools at 2 μg/ml (Mabtech), medium alone or Concanavalin A (4 μg/mL, Sigma-Aldrich). The peptides were 15-mers overlapping with 11 amino acids, covering the S1 domain of the spike S protein (amino acid 13-685); pool 1 SARS-CoV-2 S1 peptides 1–83 (83 peptides), pool 2 SARS-CoV-2 S1 peptides 84-166 (83 peptides). After 20 ± 2 h of incubation at 37 °C with 5% CO2, plates were developed as recommended by the manufacturer. Plates were analyzed using the Immunospot analyzer and software (Immunospot).

### Statistical analysis

Graphs were prepared using GraphPad Prism software (version 7). Statistical analysis was performed using Kruskal Wallis test followed by Dunn’s test for multiple comparisons (*p* < 0.05 was used to indicate significance).

## Results

### Generation of DREP vaccine constructs expressing SARS-CoV-2 spike protein

We generated DNA-launched replicons encoding either SARS-CoV-2 spike protein with an 18-aa long deletion at the cytoplasmic tail to remove an endoplasmic reticulum (ER)-retention signal^27^ or the prefusion stabilized ectodomain with a C-terminal T4 trimerization domain^19,20,22^, as candidate vaccines against SARS-CoV-2 (Fig. 1A).

Expression of spike variants from the DREP constructs was confirmed after transfection of 293T cells using Western blot and anti-spike serum. The full-length proteins, similar in size to recombinant purified spike, were detected in cell lysates for both constructs and in culture supernatant for the DREP-S^ecto^ construct encoding the spike ectodomain, while no protein product of this size was detected in cells transfected with the control DREP expressing chikungunya virus glycoproteins^23,28^ (Fig. 1B). An additional lower molecular weight band was detected in lysate and supernatant of the DREP-S transfected cells. These bands likely correspond to cleaved S1 (supernatant) and S2 (lysate) in this construct with intact furin cleavage site. In contrast, these lower molecular weight bands were absent in cells and supernatant transfected with DREP-S^ecto^ in which the furin site is mutated.

### The DREP-S and DREP-S^ecto^ vaccine candidates are immunogenic in mice

**Figure 2:**
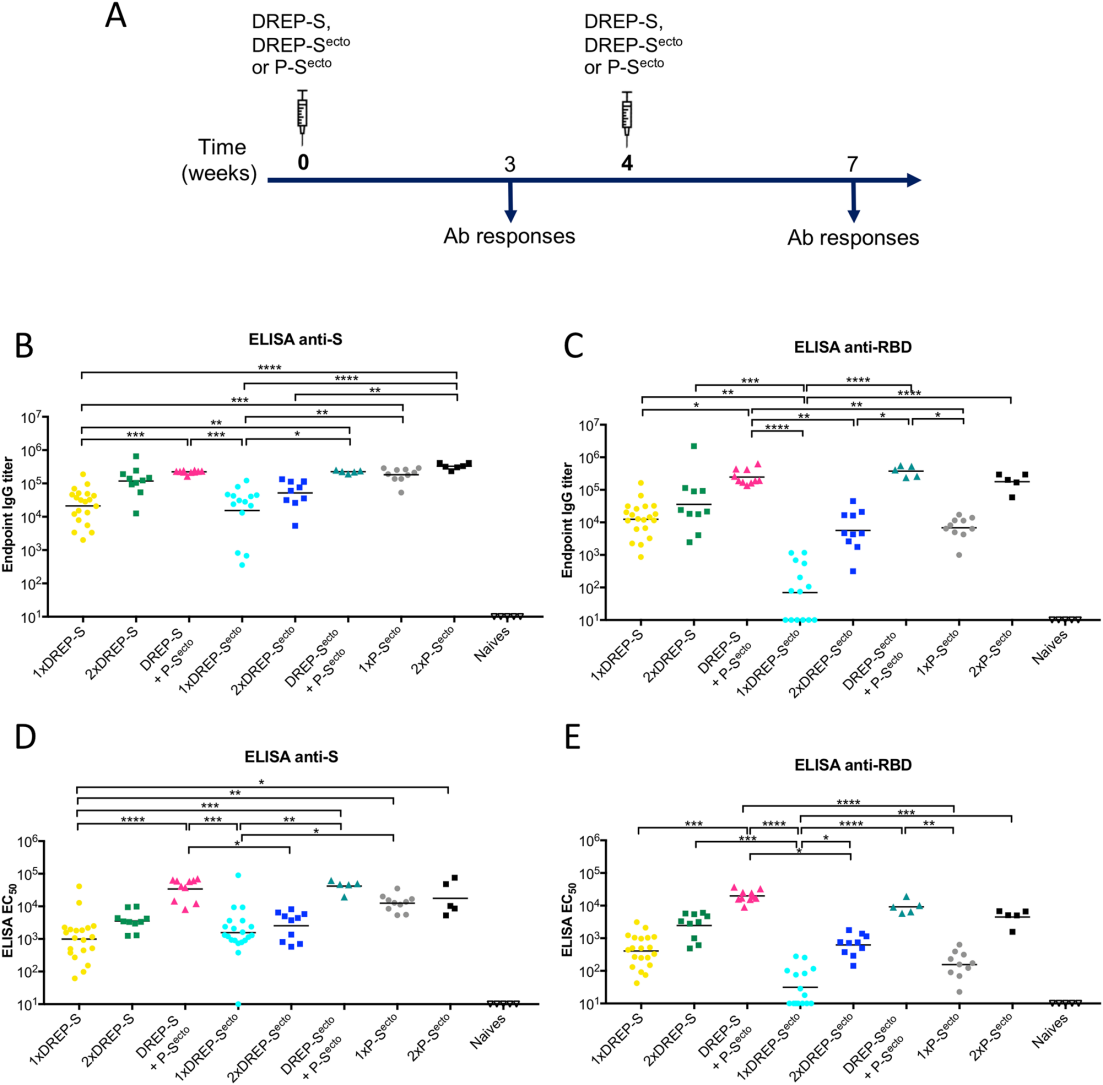
Anti-SARS-CoV-2 IgG antibody responses. (**A**) Schematic representation of the experiment schedule. C57BL/6 J mice (n = 5–20/group) were immunized once or twice with 10 μg of either DREP-S, DREP-S^ecto^ or S^ecto^ protein (P-S^ecto^) and blood samples were collected three weeks after each immunization. Individual serum samples were assayed by ELISA using plates coated with SARS-CoV-2 spike (S) or receptor binding domain (RBD) antigens (**B**,**D** and **C**,**E**, respectively). The results are presented either as endpoint antibody titers (**B**,**C**) or EC_50_ values (**D**,**E**). Statistical analysis was performed using Kruskal Wallis test followed by Dunn’s test for multiple comparisons, *p < 0.05; **p < 0.01; ***p < 0.001, ****p < 0.0001. Comparisons that were non-significant are not indicated.

To evaluate the immunogenicity of the DREP-S and DREP-S^ecto^ candidate vaccines, C57BL/6J mice were immunized with 10 μg of either DREP construct in homologous prime-boost regimens (Fig. 2A). We also performed heterologous prime-boost immunizations with either DREP construct as a prime and recombinant spike protein (designated P-S^ecto^) as a boost. For comparison, we performed one or two immunizations with 10 μg of recombinant pre-fusion stabilized spike protein (P-S^ecto^) of SARS-CoV-2 in Addavax (InvivoGen) adjuvant, a squalene-based oil in-water emulsion analogous to MF59.

A single immunization with DREP-S induced potent anti-spike antibody responses (Fig. 2B), with endpoint titers ranging from ~ 10^3^ to 5 × 10^5^ (geometric mean of 21,246) while the DREP-S^ecto^ immunization resulted in slightly lower anti-S antibody titers, ~ 10^2^–10^5^ (geometric mean of 15,365). However, there was no statistical difference between the two DREP groups after initial prime. The homologous boost immunizations augmented the anti-S endpoint antibody titers about sixfold in the DREP-S group and about threefold in the DREP-S^ecto^-immunized group (geometric mean of 117,706 and 51,891, respectively). After two immunizations, the endpoint antibody titers were about twofold higher in the DREP-S group in comparison to DREP-S^ecto^ group.

With some exceptions^29,30^, the receptor binding domain (RBD) is the major target on the SARS-CoV-2 spike protein for neutralizing antibodies^31^. Therefore, we tested the antibody responses against the receptor binding domain of SARS-CoV-2 (Fig. 2C). RBD–specific antibody responses were similar in pattern and magnitude to anti-spike antibody responses for all groups except 1xDREP-S^ecto^ and 1xP-S^ecto^, indicating that most vaccine-induced IgG binding antibodies targeted RBD epitopes. A single immunization with DREP-S elicited a strong RBD-specific IgG antibody response, ranging from ~ 10^3^ to 10^5^, and seroconversion was evident in all mice. DREP-S^ecto^ however, was less immunogenic, giving weak or undetectable responses after the initial prime. After the homologous boost, both vaccine candidates were immunogenic, inducing robust binding antibody responses to the RDB in all immunized mice. However, the responses in the DREP-S^ecto^ group were about sixfold lower (geometric mean of 5,673) than in the DREP-S-immunized mice (geometric mean of 35,892).

We next tested whether the immune responses could be further boosted by heterologous boost with the prefusion stabilized SARS-CoV-2 spike protein (P-S^ecto^). Mice that received the identical priming immunization with DREP-S or DREP-S^ecto^ are represented as single, separate groups before booster immunizations with different vaccine candidates (DREP-S/DREP-S^ecto^ or P-S^ecto^) were administered. The RBD-specific antibody titers elicited after a DREP-S prime were augmented about 20-fold by the P-S^ecto^ boost (Fig. 2B,C), compared to a sixfold increase in the homologous prime-boost regimen, demonstrating the potential to further boost the induction of binding antibodies by a DREP-S priming followed by a P-S^ecto^ boosting immunization protocol. The difference in antibody responses between the two groups were even more evident when the results were analyzed as EC_50_ values (Fig. 2D,E). In the group primed with DREP-S^ecto^, the anti-RBD antibody responses after heterologous protein boost were significantly higher than after the homologous boost (geometric mean titer of 374,030 and 4598, respectively). Notably, although homologous boost resulted in higher responses in the DREP-S group than in the group immunized with DREP-S^ecto^, the antibody levels after the heterologous boost were equally strong in both groups.

Interestingly, a single immunization with the prefusion stabilized SARS-CoV-2 spike protein (P-S^ecto^) with Addavax adjuvant induced very high anti-S endpoint antibody titers with a geometric mean of 182,000. After one immunization the titers were comparable to those of mice immunized two times with DREP-S. Given that the same S^ecto^ protein was used for the immunization and for coating the plates in the ELISA assay, this result is not surprising. Importantly, the antibody titers against the receptor binding domain (anti-RBD) induced with P-S^ecto^ were over 25-fold lower than total anti-spike antibody titers, with geometric mean of 6832. On its own, P-S^ecto^ immunization was significantly less immunogenic than DREP-S or DREP-S^ecto^ prime followed by the P-S^ecto^ boost indicating that responses were efficiently primed by the DREP vaccine candidates. Two doses of P-S^ecto^ resulted in elevated anti-S and anti-RBD titers with geometric mean of 329,086 and 177,241, respectively.

### The DREP-S and S^ecto^ vaccine candidates favor Th1 type antibody profiles

**Figure 3:**
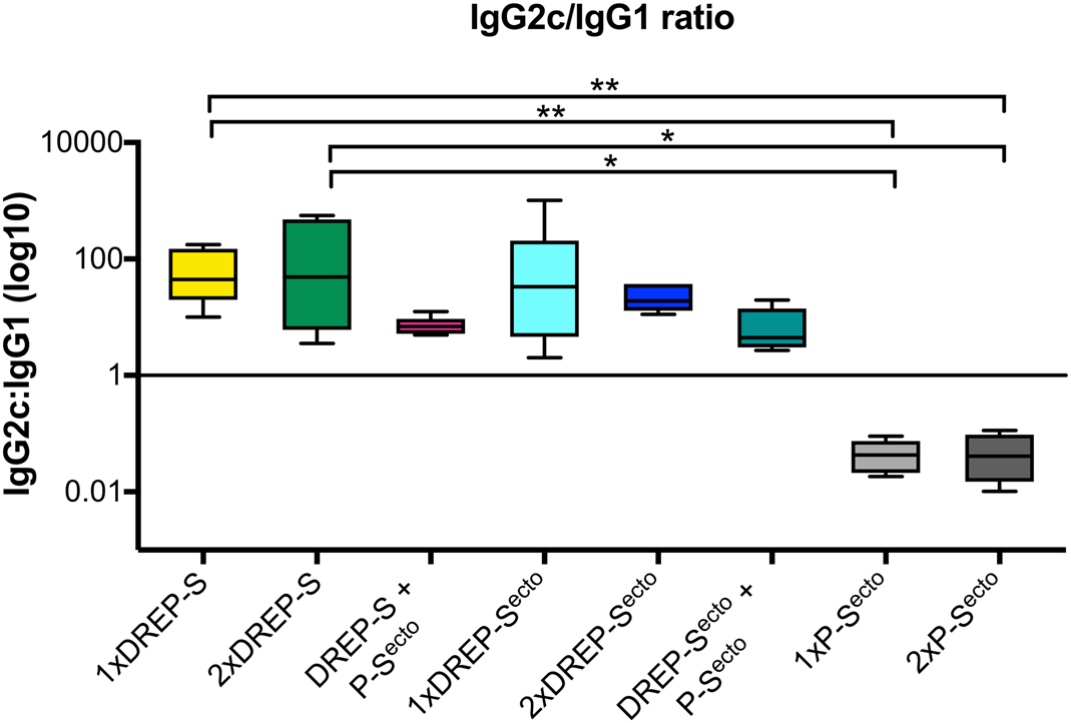
Anti-S SARS-CoV-2 antibody isotype analysis. Sera from mice immunized once or twice with DREP-S, DREP-S^ecto^ or with the S^ecto^ protein (n = 5/group) were analyzed by ELISA for anti-S^ecto^ SARS-CoV-2 antibody isotypes IgG2c and IgG1. Results are presented as IgG2c:IgG1 endpoint antibody titer ratios against spike protein. A ratio above 1 indicates a Th1 biased response. Statistical analysis was performed using Kruskal Wallis test followed by Dunn’s test for multiple comparisons, *p < 0.05; **p < 0.01.

The generation of IgG2c or IgG1 antibodies is correlated with production of cytokines characteristic for a T helper 1 or a T helper 2 profile, respectively and can be used as an indication of a predominantly Th1- or Th2-biased immune response^32^. As we showed previously^6,23,25^, DREP-based vaccines drive mostly Th1 type responses but in some cases, Th profile might partially depend on the antigen^25^. In order to determine how the DREP vaccine candidates affected Th1 and Th2 induction, we evaluated the ratio of IgG2c and IgG1 antibody titers against SARS-CoV-2 spike antigen (Fig. 3). We observed that mice immunized with one or two doses of the DREP-S or DREP-S^ecto^ constructs induced higher IgG2c antibody titers compared to IgG1, corresponding to a Th1 biased response. In contrast, mice given one or two doses of S^ecto^ protein without a DREP prime had a stronger IgG1 response compared to IgG2c response, demonstrating that protein/Addavax adjuvant skews the response to a Th2 phenotype. The anti-S^ecto^ IgG2c/IgG1 ratios were significantly different in animals immunized once or twice with DREP-S as compared to the P-S^ecto^ regimen (Kruskal–Wallis test). Interestingly, when a DREP-S or DREP-S^ecto^ prime was followed by S^ecto^ protein boost, higher IgG2c/IgG ratio induced by the DREP-S prime was maintained. However, this immunization regimen resulted in a more balanced IgG2c/IgG1 response.

### The DREP-S and DREP-S^ecto^ vaccine candidates induce neutralizing antibodies against SARS-CoV-2

**Figure 4:**
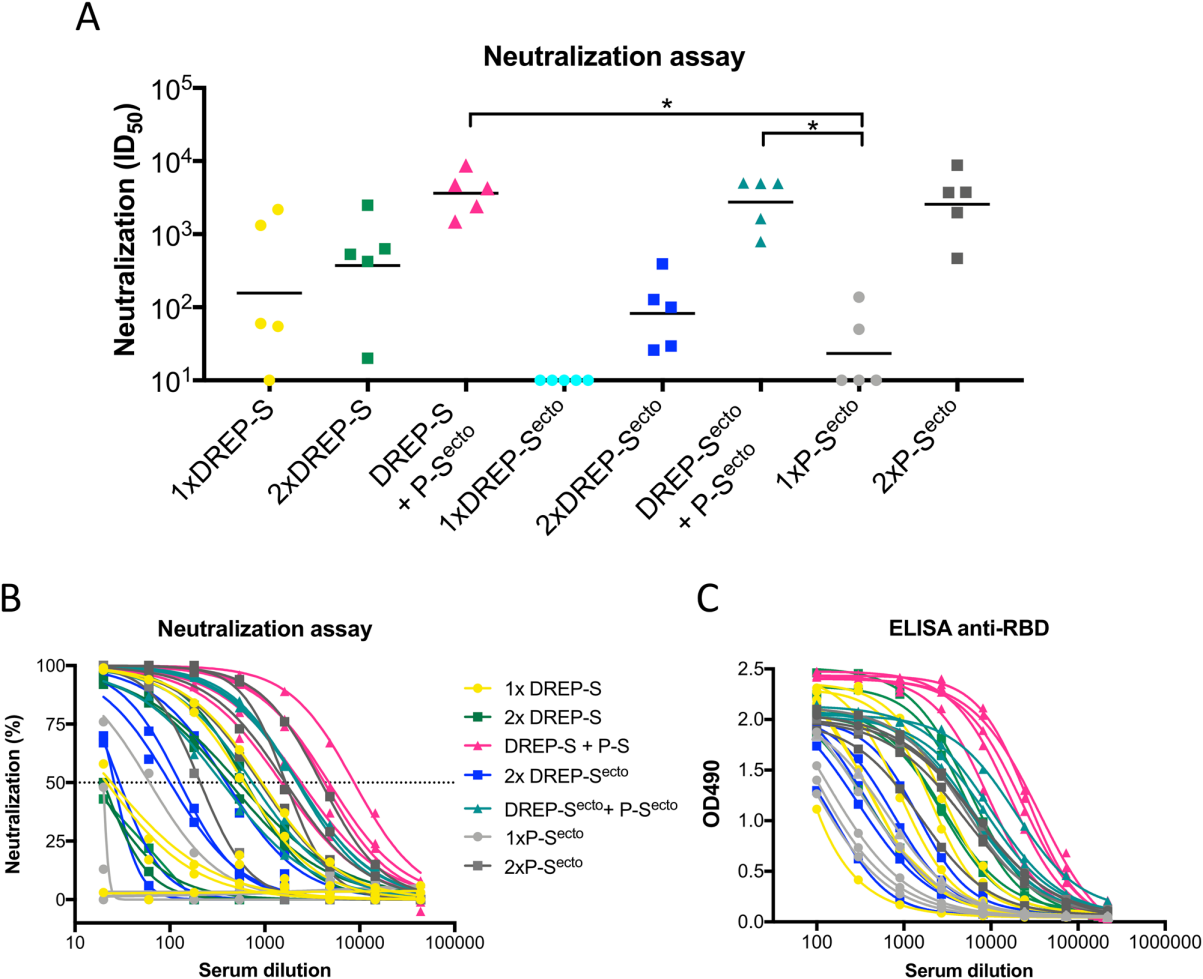
Antibody quantification and neutralization of the vaccinated mice. (**A**) Mice were immunized with 10 μg of candidate vaccines as indicated on the graph and serial dilutions of serum from vaccinated mice were assessed for neutralization of SARS-CoV-2 pseudotyped lentiviruses harboring a luciferase reporter gene. The ID_50_ titers were calculated as the reciprocal dilution where infection (RLU) was reduced by 50% relative to infection in the absence of serum. The geometric mean ID_50_ for each group is displayed. (**B**) Neutralization curves depicting percent neutralization against serum dilution. (**C**) Curves depicting OD490 readouts in anti-RBD ELISA assay detecting IgG binding antibodies in the serum of immunized mice. Across all groups, RBD-specific IgG titers correlated strongly with pseudovirus neutralization. Statistical analysis was performed comparing all groups except 1xDREP-S^ecto^ using Kruskal Wallis test followed by Dunn’s test for multiple comparisons, *p < 0.05.

To determine whether the antibody responses elicited were neutralizing, we used a SARS-CoV-2 pseudotyped virus neutralization assay^21^. In the DREP-S-immunized mice, neutralizing responses (average ID_50_ of 723) were already detectable in four of five animals after a single dose (Fig. 4A). All DREP-S-immunized mice developed neutralizing antibody responses with increased potency (average ID_50_ of 820) after the boost. In contrast, the DREP-S^ecto^-immunized mice developed neutralizing antibody responses only after the second dose, with an average ID_50_ neutralizing antibody titer of 135. Importantly, when the DREP-S or DREP-S^ecto^ prime was followed by the S^ecto^ protein boost, we observed remarkably efficient viral neutralization, with ID_50_ values ranging from 10^3^ to 10^4^. The neutralization titers in the group primed with DREP-S were similar to those observed in the group that received DREP-S^ecto^ prime, and ID_50_ values for both groups were significantly higher than in the group immunized with a single dose of S^ecto^ protein. The substantial enhancement of the virus neutralizing activity observed after the protein boost indicates that this vaccine regimen is particularly potent. A single dose of P-S^ecto^ induced neutralizing responses in two out of five mice. The titers increased after the second dose, reaching similar level of neutralizing responses as in the groups that received a heterologous prime-boost vaccination. Across all groups, pseudovirus neutralization correlated strongly with RBD-specific IgG titers (Fig. 4B,C). A summary of the relationship between neutralizing and binding antibody titers is given in Table 1.

**Table 1:** Relationship between anti-RBD binding antibody responses (EC_50_) and neutralizing antibody responses (ID_50_) against SARS-CoV-2.

Vaccine	Average EC_50_	Average ID_50_
1xDREP-S	707	725
2xDREP-S	3328	820
DREP-S + P-S^ecto^	21,356	4252
1xDREP-S^ecto^	68	0
2xDREP-S^ecto^	783	135
DREP-S^ecto^ + P-S^ecto^	10,109	3471
1xP-S^ecto^	220	43
2xP-S^ecto^	5014	3137

The animals with no detectable neutralizing antibodies (immunized once with DREP-S^ecto^) had the lowest binding antibody titers.

### Immunization with DREP-S and DREP-S^ecto^ induce SARS-CoV-2-specific T cell responses

**Figure 5:**
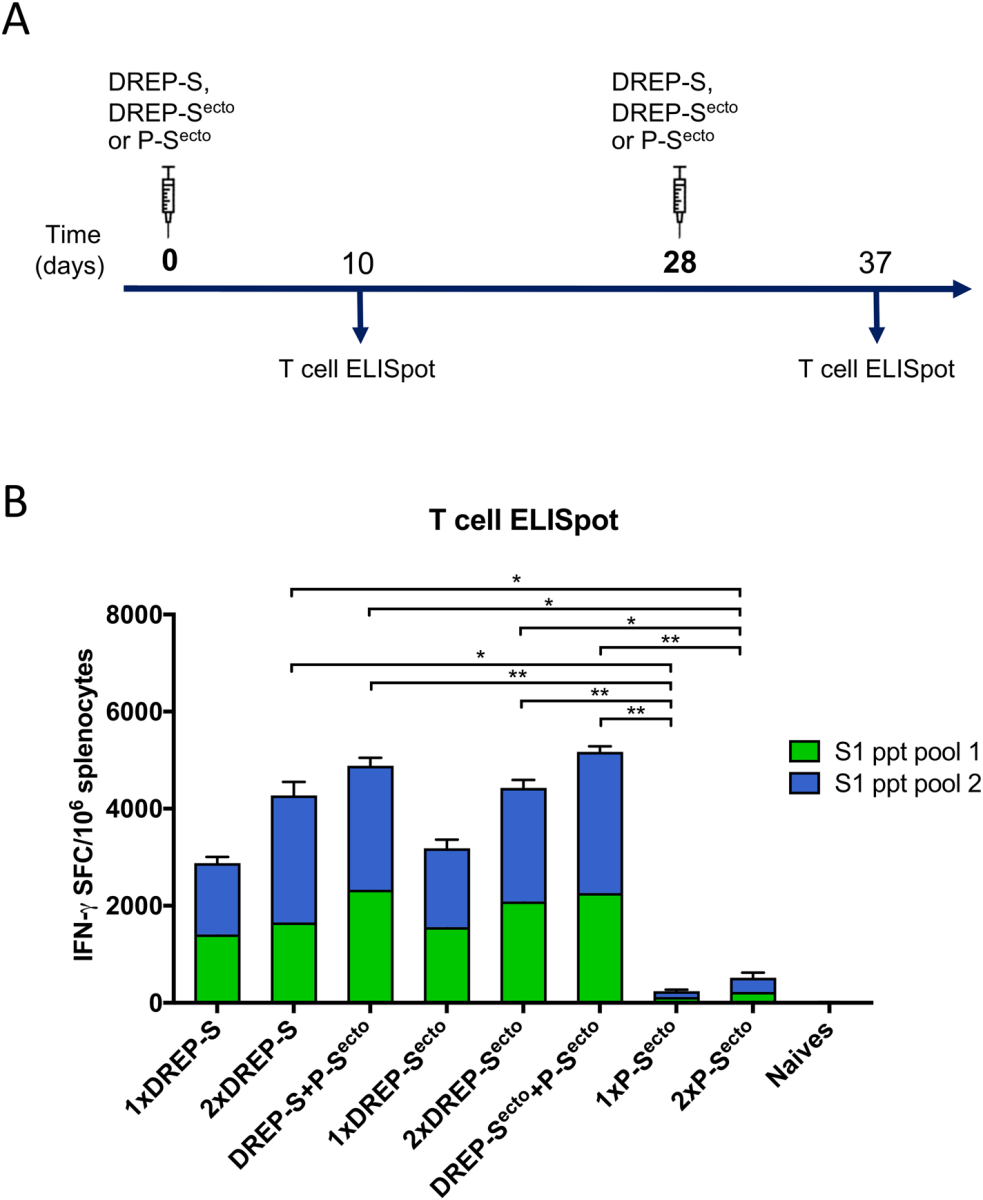
Analysis of S1-specific T cell responses. (**A**) Schematic representation of the experiment schedule. Mice were immunized on days 1 (prime) and 28 (boost) as indicated in the graph and spleens were collected 10 days after each immunization. (**B**) Quantification of IFN**-**γ secreting splenocytes upon restimulation with SARS-CoV-2 peptides. T cell responses were analyzed by ELISpot after restimulation of splenocytes with the SARS-CoV-2 S1 peptides (15-mers overlapping with 11 amino acids), covering the S1 domain of the spike glycoprotein. Pool 1, SARS-CoV-2 S1 peptides 1–83; pool 2, SARS-CoV-2 S1 peptides 84–166. Statistical analysis was performed comparing all groups except naïve controls using Kruskal Wallis test followed by Dunn’s test for multiple comparisons, *p < 0.05; **p < 0.01.

There is a growing body of evidence suggesting that T-cell responses play an important role in COVID-19 mitigation; individuals who were exposed but asymptomatic developed a robust memory T-cell response without symptomatic disease in the absence of a measurable humoral response^33–35^. Thus, we analyzed the induction of SARS-CoV-2 S1-specific T cell responses in splenocytes from mice immunized with the DREP-S and DREP-S^ecto^ vaccine candidates by IFN-γ ELISpot (Fig. 5A). We tested the reactivity to two peptide pools (15-mers overlapping by 11 amino acids), covering the S1 domain of the spike protein (amino acid 13-685) in mice that were primed and boosted with either DREP-S or DREP-S^ecto^. In addition, we analyzed T cell responses after the heterologous boost with the S^ecto^ protein. For comparison, we also evaluated T cell responses after one or two doses of S^ecto^ protein. We observed that splenocytes from vaccinated mice re-stimulated with the two pools of SARS-CoV-2 S1 peptides yielded remarkably high frequency of IFN-γ secreting cells, ranging from 1300 to 2000 SFC/10^6^ splenocytes (Fig. 5B). Interestingly, unlike the antibody responses, the T cell responses induced by a single immunization with 10 μg of DREP-S^ecto^ or DREP-S were at a very similar level. The frequencies of IFN-γ secreting cells increased with the homologous boost, ranging from 1700 to 2600 SFC/10^6^ splenocytes in the 2xDREP-S group and from 2100 to 2400 SFC/10^6^ splenocytes in the group that received two immunizations with DREP-S^ecto^. Overall, after two immunizations with either DREP-S or -S^ecto^, similar magnitudes of T cell responses were observed. The heterologous boost immunization with the P-S^ecto^ further augmented the T cell responses resulting in equally high levels of IFN-γ secreting cells (2200–3200 SFC/10^6^) in the group primed with DREP-S or DREP-S^ecto^. In contrast, immunization with spike protein generated low but detectable frequencies of IFN-γ secreting cells, which increased only slightly after the second dose. The magnitude of T cell responses elicited after one or two immunizations with P-S^ecto^ was significantly lower than those induced with one or two doses of either DREP construct.

## Discussion

The COVID-19 pandemic has placed a substantial pressure on health systems to deliver a rapidly producible and scalable vaccine. New vaccine platforms, reverse genetics, computational biology, protein engineering and gene synthesis have now enabled to produce vaccines with speed and precision^18^. This resulted in over 162 candidates undergoing preclinical development and 52 already in clinical development (WHO https://www.who.int/publications/m/item/draft-landscape-of-covid-19-candidate-vaccines). These include vaccine platforms based on DNA or RNA (Moderna^36^, CureVac, BioNTech/Pfizer^37^), adenovirus vector-based vaccines (CanSinoBIO^38^, University of Oxford/AstraZeneca^39^, Janssen Pharmaceutical Companies), inactivated vaccines (Sinovac, Wuhan Institute of Biological Products), and protein subunit vaccines (Sanofi Pasteur/GSK, Novavax^40^, Clover Biopharmaceuticals/GSK/Dynavax).

Despite promising results of early clinical trials of several vaccine candidates against SARS-CoV-2, there are still concerns regarding both safety and durability of the immune responses. Consequently, it is necessary to develop additional vaccine candidates, as different vaccine approaches and platforms may have their distinct merits, and may work synergistically in heterologous prime-boost regimens. Understanding how different vaccine platforms interact may also be essential in the context of multiple vaccine candidates and limited availability of doses. An ideal vaccine against SARS-CoV-2 would be effective after one or two immunizations, conferring long-term protection to target populations such as the elderly or immunocompromised individuals, and reducing onward transmission of the virus to contacts^39^. The benefit of developing an effective vaccine is even greater if it can be deployed in time to prevent repeated or continuous epidemics. This supports the use of genetic vaccine platforms that are rapid and straightforward to produce and with previously proven efficacy.

The alphavirus-based replicon platform technology has been developed as vaccine candidates towards many different infectious diseases, including vaccines for influenza A virus (IAV)^41^, respiratory syncytial virus (RSV)^42,43^, Ebola (EBOV), hepatitis C virus (HCV), chikungunya (CHIKV, now in phase III)^28,44^, HIV (now in phase I), human papilloma virus (HPV, now in therapeutic phase II)^45^. Given the generic design of our platform and that new constructs can be made rapidly with synthetic design of the insert, it can be readily adapted to SARS-CoV-2. Moreover, when new virus species emerge, a vaccine platform that can be rapidly adapted to emerging viruses is highly desirable.

Here we characterized and compared the immunogenicity of two SARS-CoV-2 DREP vaccine candidates in a C57BL/6J mouse model. We observed that both DREP constructs were immunogenic, inducing spike-specific antibodies with neutralizing activity as well as T cell responses. Interestingly, the DREP coding for Δ18 spike was the more potent vaccine candidate than DREP-S^ecto^, eliciting high SARS-CoV-2 specific IgG antibodies that were able to efficiently neutralize pseudotyped virus after a single immunization. The immune responses could be further increased by booster immunizations with the same construct or with a heterologous boost with recombinant prefusion stabilized trimeric SARS-CoV-2 spike protein, P-S^ecto^.

Although a correlate of protection has not yet been defined for COVID-19, neutralizing antibodies targeting different epitopes of the spike glycoprotein have been associated with protection from SARS-CoV-2 challenge in a preclinical rhesus macaque study^46^. High levels of neutralizing antibodies have also been detected in convalescent individuals^47^.

We here show that two doses of DREP-S or DREP-S^ecto^ candidate vaccines induced neutralizing antibody titers in all immunized mice. We also found strong correlations between the binding and neutralizing antibody assays, and observed that binding IgG antibody titers against SARS-CoV-2 receptor binding domain (RBD) correlated stronger with the neutralization IC_50_ than the anti-spike antibody titers. Given the role of RBD in binding to the ACE2 receptors, such strong correlation is not unexpected^48^^,^^49^. We observed that a single immunization with the DREP-S vaccine candidate induced high titers of anti-spike and anti-RBD IgG antibodies, with endpoint titers ranging from ~ 10^3^ to 10^5^. Importantly, the DREP-S-elicited antibodies were able to efficiently neutralize SARS-CoV-2 pseudotyped virus in 4 out of 5 mice. The animal that did not develop neutralizing antibodies had the lowest anti-RBD antibody titers within the group. Both binding and neutralizing antibody titers increased in response to the second immunization.

For any vaccine intended to generate antibody-mediated immunity, delivering a conformationally correct antigen that maintains the surface contours of the native virus protein and preserves the epitopes required for eliciting high-quality neutralizing antibodies is critical^18^. It has been shown previously that conformational stability of SARS-CoV-2 spike protein translates into greater immunogenicity^49–51^ and improved protein expression^52^ which is particularly advantageous for gene-based antigen delivery. In our study, however, using an insert encoding the SARS-CoV-2 spike stabilized in its pre-fusion conformation did not improve the vaccine’s immunogenicity. On the contrary, the DREP-S^ecto^ construct was less immunogenic than DREP-S coding for the spike protein without the stabilizing mutations. A single immunization with DREP-S^ecto^ elicited anti-S and anti-RBD binding antibody titers that were respectively 1 and 2–3 orders of magnitude lower than after one immunization with DREP-S, and failed to neutralize pseudotyped virus. After homologous boost with DREP-S^ecto^, all animals developed high titers of binding antibodies against both spike and RBD that also potently neutralized pseudovirus. However, the titers were almost 1 order of magnitude lower than after two immunizations with DREP-S. Interestingly, after the heterologous boost with the S^ecto^ protein, the ID_50_ titers in the group primed with DREP-S^ecto^ were at the same level as in the group primed with DREP-S. Besides stabilizing amino acids, the S^ecto^ construct lacks the transmembrane domain and the cytoplasmic tail of the spike, which in contrast to S construct, results in expression of a secreted protein only. Whether the surface presentation of the spike, combined with the soluble S1 subunit in the DREP-S construct contributes to the better immunogenicity remains speculative. However, it has long been known that presenting multiple copies of an antigen in a repetitive way, which might be the case for the membrane-anchored S protein, can induce more robust antibody immune responses than secreted immunogens^53^. This effect is considered to result predominantly from stronger activation of B cells through cross-linking of B cell receptors (BCRs)^54^, although potential impact on antigen localization and trafficking might also be important^55^. Another explanation of such result could be the targeted delivery of membrane-anchored antigens to antigen presenting cells (APC) in a concentrated form. It has been demonstrated previously that dendritic cells (DC) were > 5000 times more efficient in the uptake and presentation of antigens when delivered on the cell surface than when in secreted form^56^. By internalization of a single cell, APC can uptake multiple copies of the membrane-bound protein. On the contrary, the amount of secreted antigen captured by APC depends more heavily on the local concentration of protein^56^. In addition, membrane-anchored protein might better preserve epitopes and contours of the native spike protein. It has been observed previously that membrane-anchored variant of spike protein of MERS-CoV elicited higher antibody responses in mice^57^. Another study showed that a DNA vaccine candidate encoding a full-length spike of SARS-CoV-2 conferred better protection in respiratory tracts of rhesus macaques, in comparison to soluble S constructs^46^. Although unexpected, this result is useful for translation as it suggests the SARS-CoV-2 spike insert delivered on the DREP platform does not require any modifications to be highly immunogenic.

Given the urgency of the current pandemic, we need various vaccine technologies and delivery routes to be explored simultaneously, as different vaccine approaches and platforms may have their distinct features, stimulating different components of the immune system and might work synergistically in heterologous prime-boost regimens. In this study, we report that a heterologous boost with a pre-fusion stabilized spike protein, P-S^ecto^ increased the binding and neutralizing antibody titers and should translate into increased potency of the vaccine regimen. Different vaccine platforms may also vary in the way they stimulate the immune system^25^. While unadjuvanted protein antigens are known to induce mainly CD4 + T helper 2 cell (Th2)–type immune response^58,59^, gene-based delivery generally promotes a CD4 + T helper 1 cell (Th1)–type immune response, which has favorable antiviral properties^25^. Previous trials with veterinary vaccines against coronavirus and animal models of SARS-1 and MERS infections have raised concerns about the risk for vaccine-associated enhanced respiratory disease^20,36^. These events were associated either with vaccine antigens that generated antibodies with suboptimal neutralizing capacity and Th2-skewed immune responses or with macrophage-tropic coronaviruses prone to antibody-dependent enhancement of replication^19,20,60^. Therefore, generation of highly neutralizing antibodies and avoiding Th2-type immune responses may be helpful in reducing the risk of antibody-dependent enhancement of replication or vaccine-associated enhanced respiratory disease^18^. As expected, both DREP vaccine candidates presented in these studies promoted IgG2c over IgG1 antibody production and elicited antibody responses indicating a strong Th1 bias, which should favor cytotoxic activity. In contrast, immunization with one or two doses of recombinant S^ecto^ protein alone was characterized by higher IgG1 responses than IgG2c indicating a Th2 type response. A vaccine regimen consisted of DREP-S or DREP-S^ecto^ prime followed by the P-S^ecto^ boost resulted with more balanced Th1/Th2 responses but was still Th1 predominant.

Both humoral and cell-mediated immune responses have been associated with vaccine-induced protection against challenge or subsequent re-challenge after SARS-CoV-2 infection in recent rhesus macaque studies^49^^,^^61^ and there is mounting evidence that T-cell responses play an important role in COVID-19 mitigation^33–35^. It has been shown that individuals who were exposed but asymptomatic developed a robust memory T-cell response without symptomatic disease in the absence of a measurable humoral response^47^. We observed that the DREP vaccine candidates induced a robust cellular response against the S1 domain of the spike protein already after one immunization. The responses increased after the homologous and heterologous boost. Interestingly, there was no statistically significant difference in magnitude of T cell responses elicited by the two DREP constructs. In contrast, immunization with one or two doses of spike protein generated very low frequencies of IFN-γ secreting cells. Therefore, a vaccination regimen encompassing a prime immunization with DREP and a protein antigen boost appears to be an excellent approach to induce antigen-specific responses from both arms of adaptive immunity.

Two other alphavirus replicon vaccine candidates against SARS-CoV-2 have been shown to be highly immunogenic in vaccinated mice^62,63^ and pigtail macaques^63^. However, in contrast to our vaccine candidates, both are RNA replicons based on Venezuelan equine encephalitis virus (VEEV). As such, these vaccine candidates are less stable than their DNA-launched replicon counterpart, DREP, and both require formulation in lipid nanoparticles before immunizations, an additional step in the vaccine manufacture process.

The DREP platform evaluated in this study has the advantage that it is inexpensive, stable, easy to produce, and showed a good safety profile in phase I clinical trials when equipped with other antigen payloads. Moreover, unlike the RNA vaccine candidates it does not require a cold-chain during transportation and storage. The platform is administered as naked DNA and is suitable for rapid adaptation such that potential new viruses/threats in an emerging outbreak can be rapidly targeted^5^. Thus, for emerging pathogens like SARS-CoV-2, the DREP platform can be an efficient and cost-effective way to replace the traditional large-scale antigen production or technology platforms that require extended time for implementation. As shown in this study, DREP can be applied alone or can be combined with a recombinant SARS-CoV-2 spike protein in a remarkably efficient prime-boost regimen. Taken together, these data provide an insight into antigen design and preclinical evaluation of immunogenicity of the DREP candidate vaccines, and open for further investigation of mixed vaccine modalities as a strategy to combat SARS-CoV-2.

## Data Availability

The datasets generated during the current study are available from the corresponding author on reasonable request.
